# A Recombinant Vaccine of H5N1 HA1 Fused with Foldon and Human IgG Fc Induced Complete Cross-Clade Protection against Divergent H5N1 Viruses

**DOI:** 10.1371/journal.pone.0016555

**Published:** 2011-01-27

**Authors:** Lanying Du, Virtual Ho-Chuen Leung, Xiujuan Zhang, Jie Zhou, Min Chen, Wu He, Hai-Ying Zhang, Chris C. S. Chan, Vincent Kwok-Man Poon, Guangyu Zhao, Shihui Sun, Lifeng Cai, Yusen Zhou, Bo-Jian Zheng, Shibo Jiang

**Affiliations:** 1 Lindsley F. Kimball Research Institute, New York Blood Center, New York, New York, United States of America; 2 Department of Microbiology, University of Hong Kong, Pokfulam, Hong Kong S. A. R., China; 3 Beijing Institute of Microbiology and Epidemiology, Beijing, China; 4 Key Laboratory of Medical Molecular Virology of MOE/MOH and Institute of Biomedical Sciences, Shanghai Medical College, Fudan University, Shanghai 200032, China.; University Paris Sud, France

## Abstract

Development of effective vaccines to prevent influenza, particularly highly pathogenic avian influenza (HPAI) caused by influenza A virus (IAV) subtype H5N1, is a challenging goal. In this study, we designed and constructed two recombinant influenza vaccine candidates by fusing hemagglutinin 1 (HA1) fragment of A/Anhui/1/2005(H5N1) to either Fc of human IgG (HA1-Fc) or foldon plus Fc (HA1-Fdc), and evaluated their immune responses and cross-protection against divergent strains of H5N1 virus. Results showed that these two recombinant vaccines induced strong immune responses in the vaccinated mice, which specifically reacted with HA1 proteins and an inactivated heterologous H5N1 virus. Both proteins were able to cross-neutralize infections by one homologous strain (clade 2.3) and four heterologous strains belonging to clades 0, 1, and 2.2 of H5N1 pseudoviruses as well as three heterologous strains (clades 0, 1, and 2.3.4) of H5N1 live virus. Importantly, immunization with these two vaccine candidates, especially HA1-Fdc, provided complete cross-clade protection against high-dose lethal challenge of different strains of H5N1 virus covering clade 0, 1, and 2.3.4 in the tested mouse model. This study suggests that the recombinant fusion proteins, particularly HA1-Fdc, could be developed into an efficacious universal H5N1 influenza vaccine, providing cross-protection against infections by divergent strains of highly pathogenic H5N1 virus.

## Introduction

Influenza epidemics and pandemics caused by influenza A virus (IAV) occur frequently. The first influenza pandemic of the 21^st^ century emerged in 2009, with a novel swine-origin influenza virus (S-OIV) H1N1 as the causative agent. Originating in Mexico, it rapidly spread to North America and then globally [1]–[4]. Of more concern, however, is the continual outbreak of highly pathogenic avian influenza (HPAI) H5N1, an influenza A subtype virus circulating in poultry, which has caused hundreds of human diseases with severe morbidity and high mortality since its re-emergence in 2003 (http://www.who.int/csr/disease/avian_influenza/country/cases_table_2010_12_09/en/index.html). Although genetic analysis of H5N1 isolated from humans revealed that all genes were of avian origin, limited person-to-person transmission of H5N1 viruses was identified [5]. Now the concern is that future influenza epidemics may be caused by new virus strains derived from mutations and/or reassortments of existing influenza viruses, particularly HPAI H5N1. Therefore, the development of effective preventive and therapeutic measures against IAV, particularly avian H5N1, is urgently needed.

Vaccination is an important strategy to counteract influenza pandemics. Current efforts to develop HPAI H5N1 vaccines are mainly focused on inactivated vaccines, which have been demonstrated to be effective in animal models for protection of H5N1 infection and to induce neutralizing antibodies in about 70% of human volunteers [6]–[8]. The other H5N1 vaccine candidates, such as those based on live-attenuated virus [9], viral vectors [10], virus-like particles (VLP) [11], DNA vaccines [12] and recombinant proteins [13], have also been tested in animal models [14], [15], and even evaluated in clinical trials [16]. However, because of the rapid mutation of hemagglutinin (HA) protein of HPAI H5N1 virus, the cross-protective immunity of these vaccines is fundamentally restricted, highly limiting their potential use against divergent H5N1 viruses.

The above fact has implied that future H5N1 influenza vaccines should be developed with more immunogenic, being able to provide broader protection against various strains of H5N1 virus infections. This may be achieved by upgrading vaccine formulas with effective adjuvants for either inactivated or live-attenuated IAV vaccines [15], designing novel vaccine components that rely on conserved sequences or universal epitopes of viral proteins, such as matrix protein 2 (M2), HA, and nucleoprotein (NP) [14], [17], updating vaccine delivery systems [18], or combining viral proteins with other components [19]. The above findings represent a significant advancement towards the development of more efficacious H5N1 influenza vaccines.

Fc of IgG is considered an important fusion tag for co-expression with several viral proteins, such as receptor-binding domain (RBD) of severe acute respiratory syndrome coronavirus (SARS-CoV), in order to facilitate purification and subsequent immunogenicity of the proteins [20], [21]. For example, fusion of Fc to HIV-1 protein has also been shown to increase the immunogenicity of fusion proteins and to elicit neutralizing antibody responses [22], [23]. This is explained by the ability of Fc to promote correct folding of the fusion proteins following expression and the possibility that IgG Fc may help to enhance binding to antigen-presenting cells (APCs) and cell lines expressing Fc receptors (FcR) [22], [24]. The foldon (Fd) sequence derived from native T4 phage fibritin has been typically incorporated at the C-terminus of collagen-like protein molecules to facilitate stabilization of protein trimer or oligomer [25], [26], indicating that C-terminal Fd is essential for correct trimerization and folding of the protein [27].

In this study, we designed two recombinant influenza vaccines based on the recombinant proteins encoding HA1 of a H5N1 virus fused to either Fc of pFUSE-hIgG1-Fc2 (human IgG Fc, thereafter named Fc) or Fd plus Fc. We then tested their immune responses and cross-protection against divergent strains of H5N1 virus in a mouse model. The developed vaccine candidates were shown to induce highly potent neutralizing antibodies that protected vaccinated mice against lethal challenge of all tested strains of H5N1 virus infection.

## Results

### Detection of Expression of HA1-Fc and HA1-Fdc Proteins

**Figure 1:**
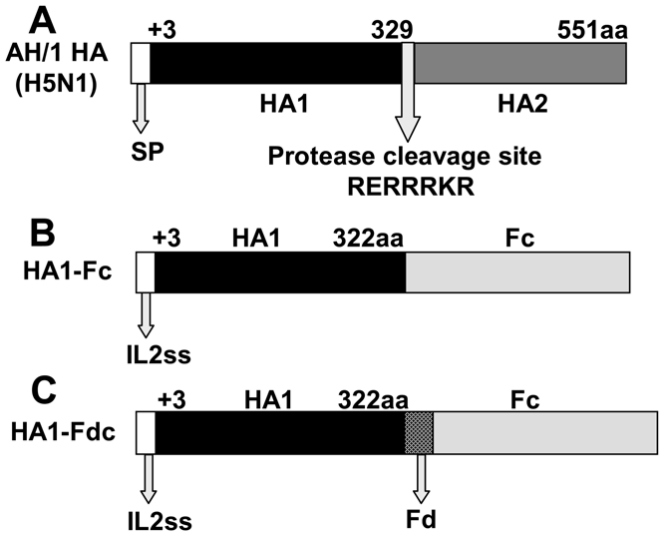
Structure of HA protein of AH/1 and construction of recombinant HA1-Fc and HA1-Fdc proteins. (A) Structure of HA protein of H5N1 AH/1 strain. The HA protein of AH/1 H5N1 virus consists of signal peptide (SP, residues 1–18), HA1 (residues 19–345, corresponding to +3-329aa) and HA2 (residues 346–567, corresponding to +330-551aa) fragments spanned by a protease cleavage site RERRRKR between HA1 and HA2. Amino acid 19 of HA1 of AH/1 corresponds to H5N1(A/Vietnam/1203/2004) amino acid +3 [28]. Schematic structures of recombinant HA1-Fc (B) and HA1-Fdc proteins (C). The original SP of HA of H5N1 virus was replaced by IL2ss signal sequence in the Fc vector, constituting HA1-Fc. HA1-Fdc was constructed by fusing Fd sequence to the C-terminus of HA1, followed by insertion into the Fc vector.

**Figure 2:**
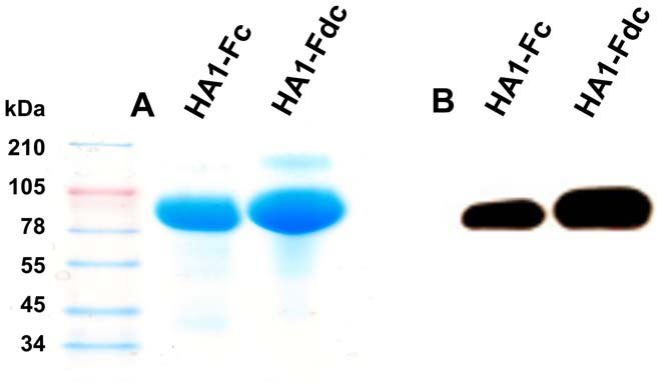
Analysis of the expression of HA1-Fc and HA1-Fdc proteins. The expression of HA1-Fc and HA1-Fdc proteins were performed by SDS-PAGE and Coomassie blue staining (A), and Western blot (B) using a HA-specific mAb. The protein molecular weight marker (kDa) is indicated on the left.

The HA1 of A/Anhui/1/2005(H5N1) (AH/1) containing 320 amino acids (residues +3-322) were fused in-frame to Fc or Fd plus Fc to form recombinant HA1-Fc and HA1-Fdc, respectively, with IL2ss of the Fc vector as the signal sequence. The recombinant proteins of HA1-Fc and HA1-Fdc will be expressed in the culture supernatant of transfected 293T cells (Fig. 1) [28], followed by purification of the proteins from the supernatant. Both proteins maintained a high level of expression, with approximately 10 mg obtained in 250 ml culture medium. After purification, the proteins were analyzed by SDS-PAGE, followed by Coomassie blue staining, and reactivity was determined using a HA-specific monoclonal antibody (mAb) developed in our laboratory [29]. As shown in Fig. 2A, one clear band was observed in samples of HA1-Fc and HA1-Fdc proteins analyzed by SDS-PAGE, indicating that highly purified proteins could be obtained from transfected culture supernatant. The purified recombinant proteins of HA1 could be recognized by the HA-specific mAb, as indicated by Western blot (Fig. 2B), revealing their high specificity to the HA of H5N1.

### HA1-Fc and HA1-Fdc Proteins Induced Strong Antibody Responses to the Homogenous HA1 Proteins and an Inactivated Heterologous H5N1 Virus

**Figure 3:**
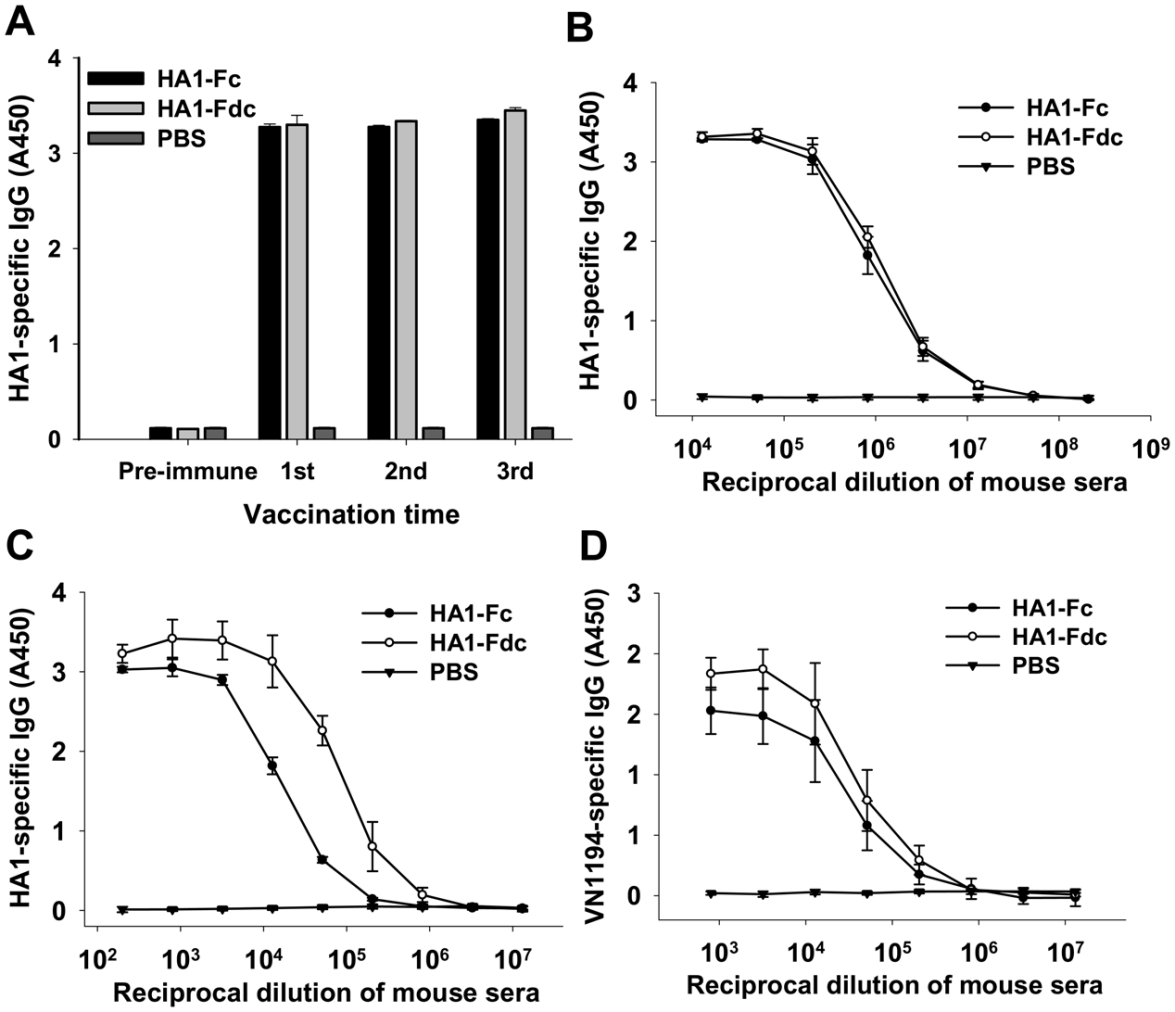
Detection of total IgG antibody responses by ELISA in HA1-Fc- and HA1-Fdc-vaccinated mouse sera. PBS was used as the negative control. (A) Reactivity of IgG antibody with HA1-Fc and HA1-Fdc proteins. The HA1-specific IgG was detected using sera (1∶3,000) from mice before (pre-immune) and 10 days after each vaccination. The data are presented as mean A450 ± standard deviation (SD) of five mice per group. Ability of IgG binding to HA1-Fc and HA1-Fdc proteins (B), HA1 protein without Fd and Fc (C), and VN/1194 inactivated H5N1 virus (D), respectively, was detected using mouse sera from 10 days post-last vaccination. The data are presented as mean A450±SD of five mice per group at various dilution points.

The antibody responses in the sera of mice vaccinated with HA1-Fc and HA1-Fdc proteins were evaluated by ELISA. As shown in Fig. 3A, both proteins induced IgG antibody responses specific to the purified HA1-Fc and HA1-Fdc proteins, with the antibody titer quickly reaching at high level after the first vaccination, then slightly increasing after each boost. An average end-point antibody titer of 1∶2.1×10^8^ was detected in mouse sera collected at 10 days post-last boost (Fig. 3B). Antibody levels were further detected by coating the ELISA plates with a HA1 protein without fusing Fc and Fd to eliminate antibody response potentially induced by the fusion tag Fc and/or Fd. As illustrated in Fig. 3C, sera of mice vaccinated with the fusion proteins, particularly HA1-Fdc, reacted strongly with this HA1 protein, reaching an end-point titer of 1∶1.3×10^7^, demonstrating that the antibody responses induced by the expressed proteins were mainly targeted the HA1 protein. It was further shown that the induced IgG antibodies could also react with an inactivated heterologous virus A/VietNam/1194/2004(H5N1) (VN/1194), having a similar end-point antibody titer of 1∶1.3×10^7^ (Fig. 3D). No IgG antibody response was detectable in the sera of control mice injected with PBS (Fig. 3).

**Figure 4:**
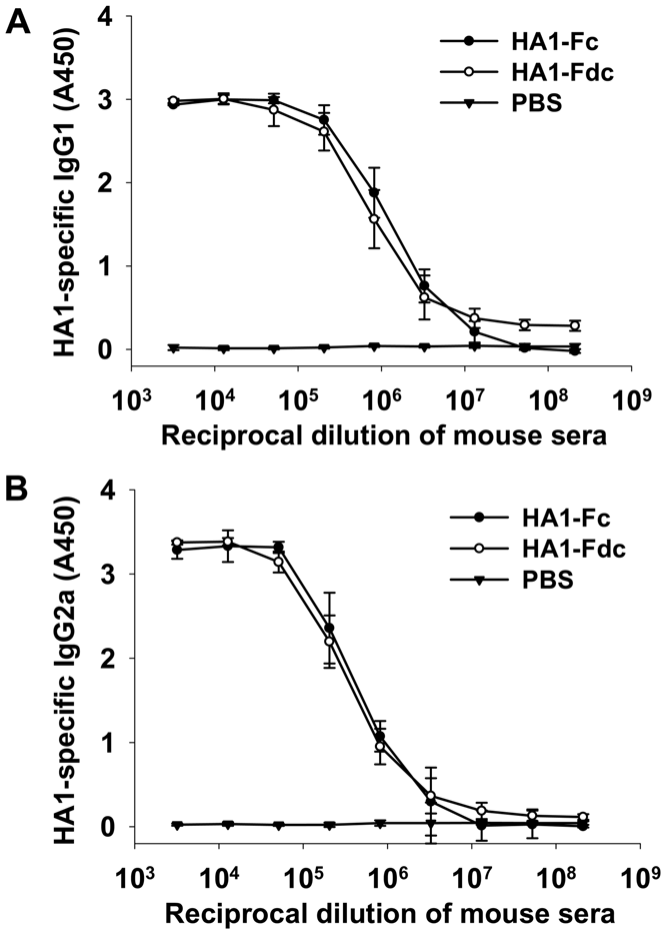
Measurement of IgG1 and IgG2a antibody titers by ELISA in HA1-Fc- and HA1-Fdc-vaccianted mouse sera. PBS was used as the negative control. Ability of IgG1 (A) and IgG2a (B) antibodies binds to the HA1 protein was detected using sera from 10 days post-last vaccination. The data are presented as mean A450±SD of five mice per group at various dilution points.

IgG1 and IgG2a subtypes induced by HA1-Fc and HA1-Fdc proteins were detected in the mouse sera collected at 10 days post-last vaccination. The results showed that both HA1-Fc and HA1-Fdc elicited similar levels of IgG1 (Th2-associated, Fig. 4A) and IgG2a (Th1-associated, Fig. 4B) antibody responses specific to the HA1 proteins, reaching an end-point antibody titer of 1∶2.1×10^8^. No IgG1 or IgG2a antibody response was found in the sera of PBS control mice (Fig. 4).

The above data suggest that expressed HA1-Fc and HA1-Fdc proteins are able to elicit high titers of antibody responses specific to the HA1 proteins of homogeneous and heterogeneous H5N1 viruses, implying their high immunogenicity in stimulating strong humoral immune responses in the vaccinated mice.

### HA1-Fc and HA1-Fdc Proteins Elicited High Titers of Neutralizing Antibodies against Infection by Different Clades of H5N1 Strains

**Figure 5:**
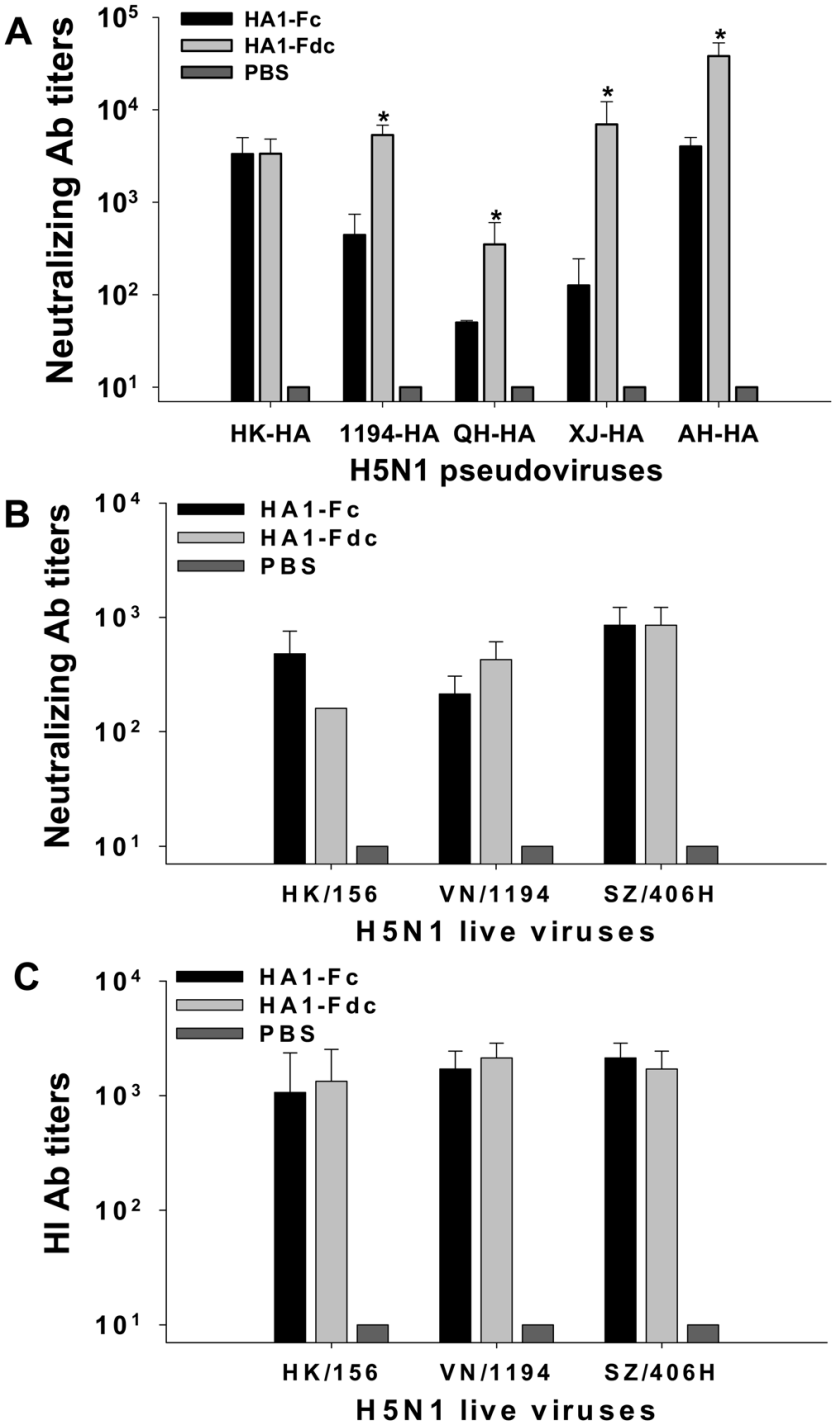
Assessment of neutralizing antibody and HI antibody titers in HA1-Fc- and HA1-Fdc-vaccinated mouse sera. Sera were collected at 10 days post-last vaccination. PBS was used as the negative control. (A) Neutralizing antibody titers against HA of heterologous (HK-HA, 1194-HA, QH-HA and XJ-HA) and homologous (AH-HA) strains of H5N1 pseudovirus. The data are presented as mean 50% neutralizing antibody titer (NT_50_) ±SD from five mice per group. * indicates significant difference (*P*<0.05) between HA1-Fc and HA1-Fdc vaccination groups. (B) Neutralizing antibody titers against heterologous strains (HK/156, VN/1194 and SZ/406H) of H5N1 live virus. The titers of neutralizing antibodies were determined as in Materials and Methods, and are presented as mean NT_50_±SD of five mice per group. (C) HI antibody titers against heterologous strains (HK/156, VN/1194 and SZ/406H) of H5N1 live virus. The HI titers were determined as the highest serum dilution indicating hemagglutinating activity, and are expressed as mean ± SD of five mice per group.

Levels of neutralizing antibodies in the vaccinated mice were evaluated in cell cultures infected with four clades (clade 0, 1, 2.2, 2.3) of H5N1 pseudoviruses [29] and three clades (clade 0, 1, 2.3.4) of H5N1 live viruses (Table 1). Fig. 5A showed that HA1-Fc and HA1-Fdc were able to induce highly potent neutralizing antibody responses in the vaccinated mice, which neutralized not only homologous AH-HA strain but also heterologous strains of H5N1 pseudoviruses, including HK-HA, 1194-HA, QH-HA, and XJ-HA. The results also demonstrated that induced antibodies could neutralize infections of heterologous strains of H5N1 live viruses, such as HK/156, VN/1194, and SZ/406H, reaching the highest titer of 1∶8.5×10^2^±3.7×10^2^ against SZ/406H H5N1 virus (Fig. 5B). In addition, these antibodies could inhibit hemagglutination of the above three H5N1 live viruses, with the average hemagglutination inhibition (HI) titer ≥1∶1.0×10^3^ (Fig. 5C). Notably, neutralizing antibodies induced by HA1-Fdc protein were greater than those induced by HA1-Fc protein, showing a significantly higher level of inhibition to infections of 1194-HA, QH-HA, XJ-HA and AH-HA H5N1 pseudoviruses (*P*<0.05). In contrast, PBS control group only elicited a background level of neutralizing and HI antibody titers in the tested H5N1 pseudoviruses and live viruses (Fig. 5).

**Table 1:** Summary of H5N1 pseudovirus and live virus strains used in the study.

Virus name	Virus strain	Virus type	Source	Clade	Pathogenicity in humans
HK-HA	A/Hong Kong/156/97	Pseudovirus	Heterologous	0	No
1194-HA	A/VietNam/1194/2004	Pseudovirus	Heterologous	1	No
QH-HA	A/Qinghai/59/05	Pseudovirus	Heterologous	2.2	No
XJ-HA	A/Xinjiang/1/2006	Pseudovirus	Heterologous	2.2	No
AH-HA	A/Anhui/1/2005	Pseudovirus	Homologous	2.3	No
HK/156	A/Hong Kong/156/97	Live virus	Heterologous	0	High
VN/1194	A/VietNam/1194/2004	Live virus	Heterologous	1	High
SZ/406H	A/Shenzhen/406H/06	Live virus	Heterologous	2.3.4	High

### HA1-Fdc Completely Protected Mice against High-Dose Lethal Challenge of Three Clades of H5N1 Strains

**Figure 6:**
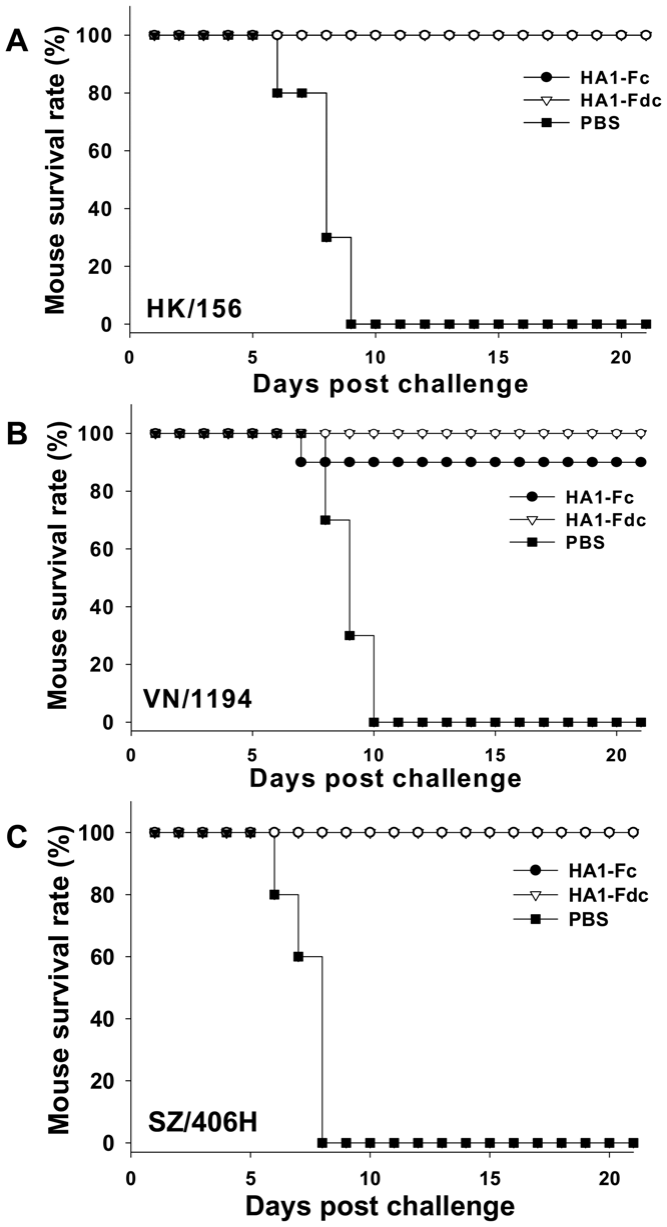
Cross-clade protection of HA1-Fc- or HA1-Fdc-vaccinated mice against lethal H5N1 virus challenge. HA1-Fc- and HA1-Fdc-vaccinated mice were challenged with 10 LD_50_ of three clades (clade 0: HK/156, clade 1: VN/1194, and clade 2.3.4: SZ/406H) of H5N1 virus. PBS was used as the negative control. Groups of 10 mice were observed for survival for 21 days post-virus challenge, and corresponding survival rates were calculated. Survival rate (%) of mice challenged with HK/156 (A), VN/1194 (B), and SZ/406H (C) H5N1 virus was shown.

To examine the cross-protective effect induced by HA1-Fc and HA1-Fdc proteins, vaccinated mice were challenged with 10 50% lethal dose (10 LD_50_) of the above three H5N1 live viruses 10–12 days post-last vaccination. All vaccinated mice survived challenge with HK/156 (Fig. 6A) and SZ/406H (Fig. 6C), suggesting that the recombinant protein-based vaccines may completely protect mice against challenges with clade 0 and 2.3.4 strains of H5N1 virus. All mice vaccinated with HA1-Fdc also survived challenge with VN/1194, whereas about 10% of HA1-Fc-vaccinated mice did not survive from the challenge with this virus (Fig. 6B). In contrast, no control mice injected with PBS survived the challenge with any of HK/156, VN/1194 and SZ/406H H5N1 viruses (Fig. 6). These results demonstrated that vaccination with two fusion proteins, particularly HA1-Fdc, could provide cross-clade protection against divergent strains of H5N1 virus infection.

### Vaccination of HA1-Fc and HA1-Fdc Proteins Reduced Virus Replication and Limited Lung Damage in the Mice Infected by Divergent Strains of H5N1 Virus

**Figure 7:**
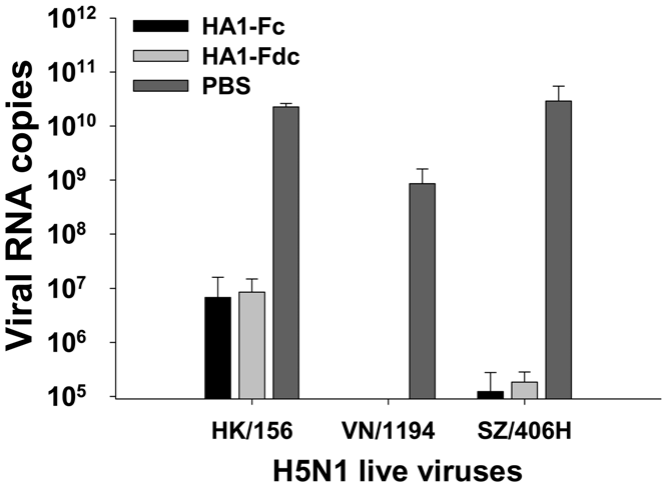
Detection of viral RNA copies by quantitative reverse-transcription PCR (Q-RT-PCR) in lung tissues of H5N1 virus-challenged mice. Titers of HK/156, VN/1194 and SZ/406H H5N1 virus were determined in lung tissues of the mice vaccinated with HA1-Fc and HA1-Fdc proteins, respectively. Mice vaccinated with PBS were used as the negative control. The data are expressed as mean ± SD of RNA copies per lung tissues from five mice per group.

The cross-protective effect elicited by HA1-Fc and HA1-Fdc proteins was further evaluated by detection of the viral load and histopathological changes in lung tissues of mice collected at day 5 post-virus challenge. Fig. 7 revealed that viral RNA was undetectable in the HA1-Fc- and HA1-Fdc-vaccinated mice challenged with VN/1194 virus, but a high level of viral RNA (8.6×10^8^±7.4×10^8^ copies/lung tissues) was detected in the control mice injected with PBS. Lung tissues of mice vaccinated with HA1-Fc and HA1-Fdc also exhibited significantly lower level of viral RNA than those of PBS control group after challenge with HK/156 and SZ/406H virus, respectively (*P*<0.05). These results suggest that the immunity induced by the recombinant fusion proteins is able to highly suppress virus replication in the vaccinated mouse lungs.

**Figure 8:**
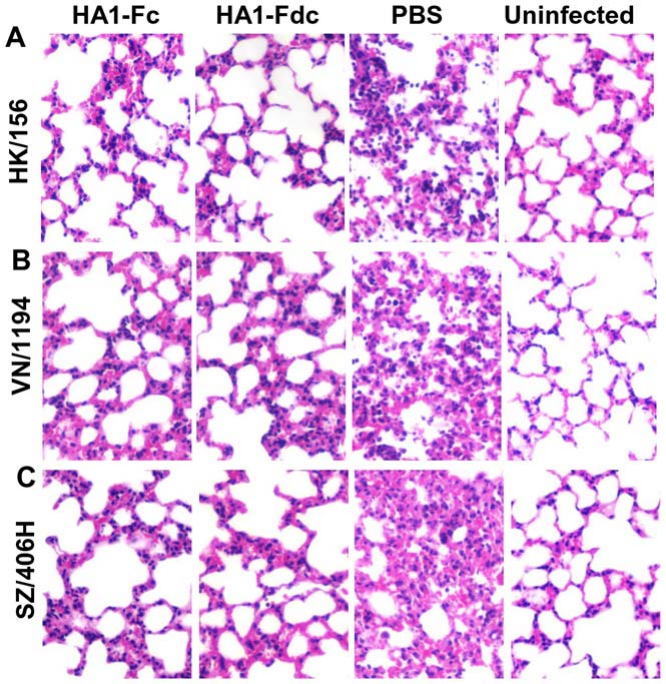
Evaluation of histopathological changes in lung tissues of H5N1 virus-challenged mice. HA1-Fc- and HA1-Fdc-vaccinated mice were challenged with 10 LD_50_ of three clades (clade 0: HK/156, clade 1: VN/1194, and clade 2.3.4: SZ/406H) of H5N1, and lung tissues were collected at day 5 post-challenge. Lung tissues from mice injected with PBS and those of uninfected mice were used as negative and normal controls, respectively. All sections of lung tissues were stained with hematoxylin and eosin (H&E) and observed under light microscope (magnification, 100×). Representative image of histopathological damage from mice challenged with HK/156 (A), VN/1194 (B), and SZ/406H (C) H5N1 virus was shown.

Examination of the H&E-stained lung tissues from virus-challenged mice demonstrated that all of the control mice injected with PBS developed a high degree of histopathological damage, including serious interstitial pneumonia and significant inflammation, which were characterized by diffused alveoli collapse, predominant lymphocyte infiltration, epithelial cell degeneration, pulmonary vascular dilatation and congestion, and focal hemorrhage and exudation. In contrast, mice receiving HA1-Fc and HA1-Fdc vaccines only presented slightly broadened interstitial spaces with little lymphocyte infiltration after challenge with all H5N1 viruses covering three different clades (Fig. 8).

## Discussion

Antibody responses provide essential immunity against infection of IAVs [30]. As the main surface protein of the virus, HA of H5N1 virus serves as an important target for induction of specific antibodies with neutralizing activity and/or protective immunity against HAPI H5N1 virus [31], [32], and it has a greater contribution than other viral proteins, such as NA, NP and M2, to the induction of neutralizing antibodies and/or protection [31], [33]. HA-based vaccines have been shown to elicit higher titers of neutralizing antibodies to prevent influenza virus infection in tested animals [14], [18], [34]. In addition, human clinical trials of the HA-containing H5N1 vaccines have revealed the production of strong and broad antibody responses after vaccine injection [35], [36]. However, current influenza vaccines have failed to provide sufficient protection against infections of rapidly mutated influenza viruses. Thus, novel strategies are urgently needed to develop vaccines potentially inducing cross-clade protection against multiple strains of influenza viruses.

Fc fragment of human IgG has been previously used as a fusion compartment to improve immunogenicity of the proposed proteins [20], [21], [23]. The underlying mechanism of Fc fusion protein-based vaccines may be partially due to that proteins with Fc could bind to cells with FcR, while the latter plays important roles in the clearance of virus infections and protection against virus challenge via FcR-mediated phagocytosis [37].

Indeed, our study has demonstrated that both Fc-containing influenza vaccine candidates, HA1-Fc and HA1-Fdc, were able to elicit strong HA1-specific antibody responses and to neutralize divergent strains of homologous and heterogeneous H5N1 pseudoviruses, as well as heterologous live virus. Importantly, both HA1-Fc and HA1-Fdc proteins could elicit potent protective immunity in the immunized mice against challenges of at least three heterogeneous strains of H5N1 viruses.

In addition to Fc, another component, Fd, could also be considered as a fusion partner to help increasing immunogenicity of recombinant vaccines. This is because that Fd, a trimeric motif, can maintain the native trimeric conformation of viral envelope proteins, thus enhancing vaccine efficacy [25], [32]. For example, a recombinant protein expressing HIV-1 gp41 NHR fused with Fd exhibited trimeric conformation and possessed potent anti-HIV-1 activity against a broad spectrum of HIV-1 strains [38].

In this study, we found that HA1-Fdc induced higher neutralizing antibodies than HA1-Fc against several strains of pseudoviruses and VN/1194 H5N1 live virus (Fig. 5), and stronger protective immunity than HA1-Fc against challenge with H5N1 strain VN/1194 (Fig. 6B). These results confirm that Fd plays a role in maintaining the native conformation of neutralizing epitopes in the oligomeric immunogen to induce stronger immune response in the vaccinated animals [38].

In summary, we have designed and constructed two recombinant influenza vaccine candidates by fusion of viral HA1 fragment, a major target of H5N1 influenza vaccines, with Fc of human IgG and/or Fd. In this proof-of-concept study, we first demonstrated that the fusion of viral HA1 antigen, with either Fc alone or both Fd and Fc, was able to induce cross-neutralizing antibodies strong enough against various subtypes of H5N1 virus, which, in turn, could provide cross-protection against infections of different clades of H5N1 virus in cell cultures *in vitro* and in mouse models *in vivo*. Thus, our data suggest the possibility of developing universal H5N1 influenza vaccines by using such innovative strategies as fusion of viral antigens with Fd and Fc, as reported in this study. Future studies will be needed to elucidate the mechanism of the cross-protective immunity induced by the proposed candidate vaccines.

## Materials and Methods

### Ethics Statement

The study of animals was carried out in strict accordance with the recommendations in the Guide for the Care and Use of Laboratory Animals of the National Institutes of Health and University of Hong Kong. All experimental protocols followed the standard operating procedures of the approved biosafety level-3 (BSL-3) animal facilities and were approved by the Committee on the Use of Live Animals in Teaching and Research of the University of Hong Kong (CULATR Ref No.: 746-03) [39].

### Construction, Expression, and Purification of Recombinant HA1-Fc and HA1-Fdc Proteins

The genes encoding HA1 of H5N1 were amplified by PCR using codon-optimized full-length HA of AH/1 (accession No. ABD28180) as the template and inserted into the Fc expression vector (InvivoGen, San Diego, CA) encoding the HA1-Fc vaccine. The Fd sequence derived from bacteriophage T4 fibritin was fused to the C-terminus of HA1 sequences by PCR with overlapping primers, followed by insertion into the Fc expression vector to encode the HA1-Fdc vaccine (Fig. 1). The recombinant HA1-Fc and HA1-Fdc proteins were expressed as previously described [40] with some modifications. Briefly, the sequence-confirmed recombinant plasmids were transfected into 293T cells (ATCC, Manassas, VA) seeded 24 h prior to transfection, using the calcium phosphate method. Culture medium was replaced by fresh OPTI-MEM I Medium (Invitrogen, Carlsbad, CA) 10 h later, and supernatant was collected 72 h post-transfection. The recombinant HA1-Fc and HA1-Fdc proteins in the supernatant were purified by Protein A affinity chromatography (GE Healthcare, Piscataway, NJ) according to the manufacturer's instructions.

### Western Blot

The purified HA1-Fc and HA1-Fdc proteins were analyzed by SDS-PAGE and Western blot as previously described [41] using an anti-HA mAb developed in our laboratories [29]. Briefly, purified proteins (10 µg) were separated by 10–20% Tricine gels (Invitrogen) and transferred to nitrocellulose membranes. After blocking overnight at 4°C, the blots were incubated with HA-specific mAb (1∶1,000) for 1 h at room temperature. After three washes, the blots were then incubated with horseradish peroxidase (HRP)-conjugated goat anti-mouse IgG (1∶5,000, Invitrogen) for 1 h at room temperature. Signals were visualized with ECL Western blot substrate reagents and Amersham Hyperfilm (GE Healthcare).

### Mouse Vaccination and Virus Challenge

Female BALB/c mice at 6–8 weeks were kept in BSL-3 housing and given access to standard pellet feed and water ad libitum. Mice (45 mice/group) were subcutaneously (s.c.) primed-vaccinated with 20 µg/mouse of purified HA1-Fc and HA1-Fdc proteins re-suspended in PBS in the presence of Sigma Adjuvant System (SAS, Sigma, St. Louis, MO) and boosted twice with 10 µg/mouse of immunogen containing SAS at 3-week intervals. Control mice were s.c. injected with the same volume of PBS-SAS. Sera were collected before immunization and 10 days post-each vaccination to detect HA and neutralizing antibodies. Mice were intranasally (i.n.) challenged with 10 LD_50_ of three clades of H5N1 virus, i.e., clade 0: HK/156, clade 1: VN/1194, and clade 2.3.4: SZ/406H, respectively (15 mice/group), at 10–12 days after the last vaccination. Infected mice were observed daily for 21 days or until death. Five mice/group were sacrificed on day 5 post-challenge, and lung samples were collected for virological and histopathological detection.

### ELISA

The antibody response was evaluated by ELISA in collected mouse sera as previously described [42]. Briefly, 96-well ELISA plates were pre-coated respectively with recombinant HA1-Fc and HA1-Fdc fusion proteins, HA1 protein without Fd and Fc, and inactivated H5N1 virus (VN/1194) overnight at 4°C and blocked with 2% non-fat milk at 37°C for 2 h. Serially diluted mouse sera were added to the plates and incubated at 37°C for 1 h, followed by four washes. Bound antibodies were incubated with HRP-conjugated goat anti-mouse IgG, IgG1 (Invitrogen), and IgG2a (Bethyl Laboratories, Montgomery, TX) for 1 h at 37°C. The reaction was visualized by substrate 3,3′,5,5′-tetramethylbenzidine (TMB) (Invitrogen) and stopped by 1N H_2_SO_4_. The absorbance at 450 nm (A450) was measured by ELISA plate reader (Tecan, San Jose, CA).

### Pseudovirus Neutralization Assay

The titer of neutralizing antibodies was detected in mouse sera by H5N1 pseudovirus neutralization assay as previously described [29]. Briefly, 293T cells were co-transfected with a plasmid encoding Env-defective, luciferase-expressing HIV-1 genome (pNL4-3.luc.RE) and each of the plasmids encoding HA of homologous AH-HA and heterologous HK-HA, 1194-HA, QH-HA and XJ-HA, respectively, using the calcium phosphate method. Exogenous bacterial neuraminidase (NA) (Sigma) was added 24 and 48 h later, and supernatants were harvested 72 h post-transfection for single-cycle infection. The pseudovirus-containing supernatants were incubated with serially diluted mouse sera at 37°C for 1 h before adding to 293T cells. Fresh medium was added 24 h later, followed by lysing cells 72 h later using cell lysis buffer (Promega, Madison, WI) and transferring the lysates into 96-well luminometer plates. Luciferase substrate (Promega) was added to the plates, and relative luciferase activity was determined in an Ultra 384 luminometer (Tecan). The neutralization of HA pseudovirus was calculated [43] and presented as NT_50_.

### H5N1 Virus Neutralization Assay

Titers of neutralizing antibodies of vaccinated mice were detected by neutralization assay as described previously [39], [44]. Briefly, serial two-fold diluted mouse sera were respectively mixed with 20 plaque forming units (PFU) of HK/156, VN/1194 and SZ/406H (H5N1) and incubated at 37°C for 1 h before adding to MDCK cells. Medium was replaced with fresh DMEM 1 h later, and cell culture was continued for 72 h at 37°C. Viral cytopathic effect (CPE) was observed daily and recorded on day 3 post-infection. The neutralizing antibody titer was determined based on the highest dilution of each serum, which completely suppressed CPE induced by the virus in >50% of the wells.

### HI Assay

HI assay was carried out as previously described [44]–[46] with some modifications. Briefly, serial dilutions of mouse sera were incubated with equal volumes of HK/156, VN/1194 and SZ/406H H5N1 virus, respectively, for 1 h at room temperature, followed by addition of equal volumes of 0.5% chicken red blood cells for 30 min at room temperature. The HI antibody titers were expressed as the highest serum dilution that completely inhibited hemagglutinating activity.

### Virological Tests

The viral RNA copies in lung tissues were quantified by Q-RT-PCR as previously described [39]. Briefly, total RNA in lysed lung tissues was extracted by using RNeasy Mini kit (Qiagen, Valencia, CA) and reverse-transcribed to cDNA by using applied SuperScript II Reverse Transcriptase (Invitrogen). Viral cDNA was synthesized by Superscript RT II (Invitrogen) using Uni12 primer (AGCAAAAGC). Real-time PCR was performed on the LightCycler 480 system (Roche Applied Sciences) using SYBR Green I Master (Roche) with gene-specific primer pairs (for HK/156, forward primer: 5′-TGTCAAGAAAGGAGACTCAGC-3′, reverse primer: 5′-ACCATCTACCATTCCCTGC-3′; for VN/1194, forward primer: 5′-ATACACCCTCTCACCATCGG-3′, reverse primer: 5′-ACCATCTACCATTCCCTGCC-3′; for SZ/406H, forward primer: 5′-ATACACCCTCTCACCATCGG-3′, reverse primer: 5′-ACCATCTACCATTCCCTGC-3′) targeting the H1 gene of different strains of H5N1 virus. The pcDNA3.1 plasmid, which contains the cloned H1 gene of the virus, was applied as the standard.

### Histopathological Analysis

The lung tissues of challenged mice were immediately fixed in 10% formalin and embedded in paraffin wax. Sections 4–6 µm in thickness were made and mounted on slides. Histopathological changes caused by H5N1 virus infection were examined by H&E staining and viewed under light microscope as previously described [39], [47].

### Statistical Analysis

Values were presented as mean with SD. Statistical significance among different vaccination groups was calculated by Student's *t* test using *Stata* statistical software. *P* values less than 0.05 were considered significant.
